# The Smc5-Smc6 heterodimer associates with DNA through several independent binding domains

**DOI:** 10.1038/srep09797

**Published:** 2015-05-18

**Authors:** Marc-André Roy, Thillaivillalan Dhanaraman, Damien D’Amours

**Affiliations:** 1 Institute for Research in Immunology and Cancer, and Département de Pathologie et biologie cellulaire, Université de Montréal P.O. Box 6128, Succursale Centre-Ville Montréal, QC, H3C 3J7, Canada

## Abstract

The Smc5-6 complex is required for the maintenance of genome integrity through its functions in DNA repair and chromosome biogenesis. However, the specific mode of action of Smc5 and Smc6 in these processes remains largely unknown. We previously showed that individual components of the Smc5-Smc6 complex bind strongly to DNA as monomers, despite the absence of a canonical DNA-binding domain (DBD) in these proteins. How heterodimerization of Smc5-6 affects its binding to DNA, and which parts of the SMC molecules confer DNA-binding activity is not known at present. To address this knowledge gap, we characterized the functional domains of the Smc5-6 heterodimer and identify two DBDs in each SMC molecule. The first DBD is located within the SMC hinge region and its adjacent coiled-coil arms, while the second is found in the conserved ATPase head domain. These DBDs can independently recapitulate the substrate preference of the full-length Smc5 and Smc6 proteins. We also show that heterodimerization of full-length proteins specifically increases the affinity of the resulting complex for double-stranded DNA substrates. Collectively, our findings provide critical insights into the structural requirements for effective binding of the Smc5-6 complex to DNA repair substrates *in vitro* and in live cells.

To ensure organism fitness and genetic inheritance, cells must maintain their genomic stability during proliferation. Genome integrity relies on several cellular pathways that together orchestrate key aspects of chromosome biogenesis, such as DNA repair, DNA replication and chromosome segregation^1^. DNA repair pathways play a key role in the preservation of chromosome integrity when cells experience genotoxic lesions^2^, whereas the replication and segregation pathways facilitate faithful duplication and transmission of the genome under normal proliferative conditions^3^,^4^. Members of the Structural Maintenance of Chromosome (SMC) family of proteins are central effectors of the segregation and DNA repair machineries, and as such contribute to critical activities required for the maintenance of genome stability.

SMC proteins are found in all domains of life^5^. Prokaryotic genomes encode a single SMC protein that operates as a homodimer. In contrast, eukaryotes express at least 6 SMC family members. Each SMC protein interacts with one other SMC family member, as well as with additional non-SMC elements to form 3 large complexes: the cohesin, the condensin and the Smc5-6 complexes^6^,^7^,^8^. The cohesin and condensin complexes play key roles in sister chromatid cohesion and chromosome condensation, respectively^7^,^8^. They are also involved in DNA repair. In particular, cohesin is implicated in DNA double-strand break (DSB) repair, whereas condensin is involved in DNA single-strand break repair^9^,^10^. The exact functions of the Smc5-6 complex are not completely understood, but include important roles in DNA repair by homologous recombination, restart of collapsed replication forks, maintenance of telomeres homeostasis, and ribosomal DNA (rDNA) stability^6^,^11^.

Inactivation of the Smc5-6 complex in *Saccharomyces cerevisiae*, *Schizosaccharomyces pombe*, chicken and human cells leads to faulty homologous recombination between sister chromatids^12^. Genetic analyses place the Smc5-6 complex in the same pathway as cohesin for DSB repair^12^,^13^. The function of the cohesin complex is to maintain proximity between sister chromatids^10^. To accomplish this function, two subunits of the cohesin complex must be sumoylated (Scc1 and Scc3), and the enzyme responsible for this sumoylation is the Nse2/Mms21 component of the Smc5-6 complex^13^. Another function of the Smc5-6 complex during DSB repair occurs specifically at the rDNA locus, where the complex antagonizes the activity of Rad52 in repair reactions^14^. Indeed, when DSBs are formed at the rDNA locus (in the nucleolus), homologous recombination is initiated but completion is prevented because Rad52 is excluded from the nucleolus in a Smc5-6-dependent manner^14^. This process relies on Rad52 sumoylation, but does not seem to involve the sumo ligase activity of Nse2/Mms21. In addition to its Rad52-specific DNA repair role in the nucleolus, the Smc5-6 complex is also required for completion of rDNA locus replication during S phase^15^. The exact function of the Smc5-6 complex during this process is unknown. Finally, the Smc5-6 complex is implicated in telomere homeostatis via the alternative lengthening of telomeres (ALT) pathway^16^. The complex regulates this process by Nse2/Mms21-dependent sumoylation of shelterin/telosome components. This post-translational modification promotes the recruitment of telomeres to promyelocytic leukemia bodies, thereby stimulating the ALT pathway^16^.

The Smc5-6 complex, like the cohesin and condensin complexes, must interact with DNA to accomplish its functions. It is therefore important to understand the nature of its DNA-binding activity to better understand how Smc5-6 functions are promoted in cells. It has been established that cohesin and condensin can associate with DNA in a topological manner^17^,^18^. This binding mode allows one or two pieces of DNA to enter into a ring formed by the cohesin or condensin complexes. This ring-like shape is formed via a ternary complex composed of distinct SMC proteins and a member of the kleisin family of proteins^19^,^20^. SMC proteins interact with the kleisins via their ATPase domains, whereas additional non-SMC components associate with the tripartite ring and provide ancillary functions to their respective complexes^6^,^7^,^8^. It is unclear whether a topological mode of DNA binding –like that of cohesin and condensin– would be consistent with Smc5-6 complex functions in DSB repair. Indeed, the ability of SMC ring complexes to slide along DNA molecules during the repair process might allow the Smc5-6 complex to “fall off” or dissociate from DNA at the position of the DSB^17^. If this were to occur, cells would not only lose the SMC-enforced proximity between damaged and undamaged DNA molecules, but also the presence of the Smc5-6 complex at the site of the lesion, which is unlikely to promote effective DNA repair. It is thus conceivable that Smc5-6 proteins could maintain physical proximity between distinct chromosomal DNA regions via a non-topological mechanism, as recently observed with RecN, a bacterial SMC-like protein^21^.

We previously showed that Smc5 and Smc6 monomers can bind to nucleic acids with a clear preference for single-stranded DNA (ssDNA)^22^,^23^. We are now interested in defining which regions of Smc5 and Smc6 molecules confer this DNA-binding activity. To answer this question, we divided the SMC proteins according to their characteristic parts –hinge, coiled-coil and ATPase head domains– and characterized the biochemical properties of these functional domains. We reveal herein the existence of two distinct DNA-binding domains on each SMC protein. Moreover, we show that these DNA-binding domains have a DNA substrate preference similar to that of full-length Smc5 and Smc6, and that dimerization of Smc5-6 modulates the DNA-binding properties of the complex.

## Results

### Generation of Smc5-6 heterodimers

**Figure 1:**
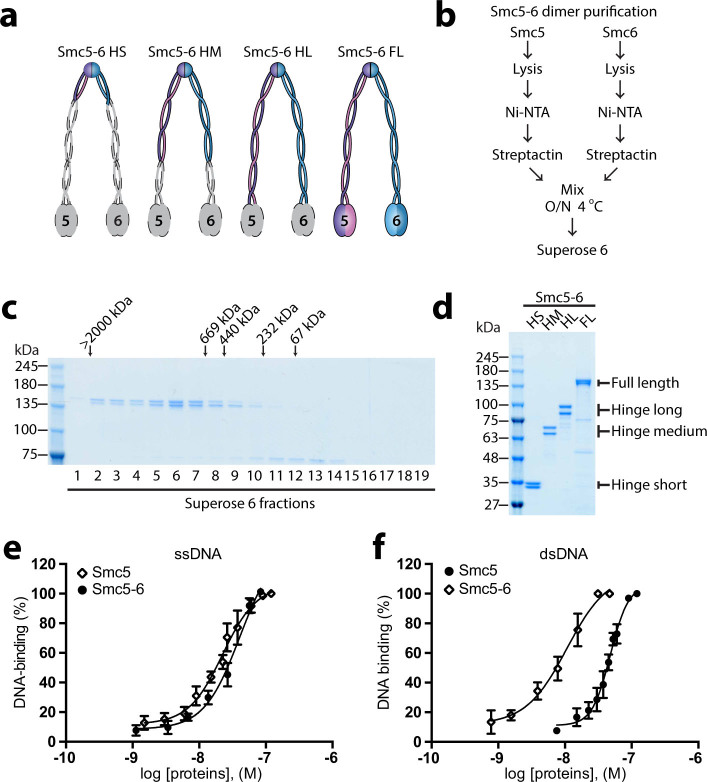
Purification and DNA-binding activity of Smc5-6 heterodimers. (a) Schematic representation of the Smc5-6 fragments and full-length proteins used in this study. The colored parts of the schematics indicate the sections of the proteins that are included in each Smc5-6 heterodimer variant. (b) Schematic representation of the Smc5-6 FL purification procedure. (c) Elution profile of the Smc5-6 FL heterodimer from the Superose 6 column. The peak positions where molecular weight standards eluted from the gel exclusion column are marked on the top of the gel. (d) The purity of the Smc5-6 heterodimers used in this study is shown after separation by SDS-PAGE and staining with Coomassie brilliant blue. (e) Association of the Smc5-6 FL heterodimer to ssDNA in a saturation binding experiment. DNA-binding was measured by EMSA with increasing concentration of Smc5-6 proteins, as previously performed^22^. The free DNA and DNA-bound forms were quantified and plotted on the graph as a percentage of the Smc5-6 heterodimer DNA-binding activity. Each dataset is the mean ± standard error from three independent experiments. For comparison, we included previously published data for Smc5 monomer binding to ssDNA^22^. (f) Association of the Smc5-6 FL heterodimer to dsDNA in a saturation binding experiment. The reactions were performed as described above, except that a duplex DNA substrate was used.

To better understand how Smc5 and Smc6 function *in vivo*, we first wanted to determine the minimal regions of these proteins that are necessary and sufficient for binding to DNA. To this end, we divided each SMC protein into three distinct regions corresponding to the domains characteristic of SMC proteins; namely the hinge, the coiled-coil and the ATPase head domains^5^. In addition to the monomeric ATPase head domains (described below), we generated four different Smc5-Smc6 heterodimer complexes: the hinge short (HS; Smc5 residues 428-to-675, Smc6 residues 486-to-732), the hinge medium (HM; Smc5 residues 300-to-803, Smc6 residues 350-to-868), the hinge long (HL; Smc5 residues 215-to-885, Smc6 residues 260-to-970), and full-length proteins (FL). Figure 1a shows a schematic representation of the different heterodimers we created (with coloured portions corresponding to the region included in each construct). The FL Smc5-6 proteins were purified from yeast as described previously^22^,^23^, whereas the protein fragments were overexpressed in bacteria either in combination or individually, and later reconstituted to form the heterodimers by sequential purification using affinity tags unique to each subunit. Specifically, all dimers were purified using a combination of nickel-chelate and *Strep*-Tactin® chromatography. Size exclusion chromatography on Superose 6 or 12 columns was performed as the final purification step to confirm the molecular mass and stability of the reconstituted complexes (Fig. 1c). The Smc5-6 FL heterodimer eluted from the exclusion column over a range of fractions corresponding to a higher molecular mass than predicted from sequence alone (~267 kDa for the heterodimer), a behaviour that is typical of SMC family proteins and that reflects both the elongated shape and the diversity of configuration of coiled-coil arms in SMC complexes^24^,^25^. Using the procedures described above, all heterodimers were purified to ≥ 90% homogeneity, as judged by Coomassie brilliant blue-staining after SDS-PAGE (Fig. 1d).

### Effects of dimerization on Smc5-6 DNA-binding activity

Next, we determined the relative affinities of the Smc5-6 heterodimers for ssDNA and dsDNA. Our initial analysis focused on the full-length (FL) version of the Smc5-6 heterodimer since DNA-binding activity is mostly or entirely conferred by SMC components in several complexes of this family^26^,^27^. We generated DNA binding isotherms from saturation binding experiments using the electrophoretic mobility shift assay (EMSA) and increasing concentrations of Smc5-6 heterodimer (*i.e*., as previously performed with the monomeric proteins^22^,^23^). The dissociation constant (*K_d_*) of Smc5-6 for ssDNA and dsDNA substrates was then calculated from saturation binding curves. The data that we previously obtained for Smc5 alone are included on the same graph for comparison^22^. As expected, the affinity for ssDNA was similar between the Smc5-Smc6 heterodimer and Smc5 monomer (*i.e*., 40 ± 3 nM *vs*. 22 ± 3 nM; Fig. 1e). In striking contrast, the heterodimer and monomer versions differed significantly in their affinity for dsDNA. Indeed, the formation of the heterodimer significantly increased the affinity of Smc5-6 FL for dsDNA relative to the Smc5 monomer alone (*i.e*., 10 ± 2 nM *vs*. 48 ± 3 nM; Fig. 1f). These results indicate that dimerization positively affect the binding of the Smc5-6 complex to dsDNA substrates. This observation is physiologically important since the Smc5 protein has been shown to function outside of the Smc5-6 holoenzyme during mitosis^28^, a period when the other components of the complex are also excluded from chromosomes *in vivo*^29^,^30^,^31^,^32^.

**Figure 2:**
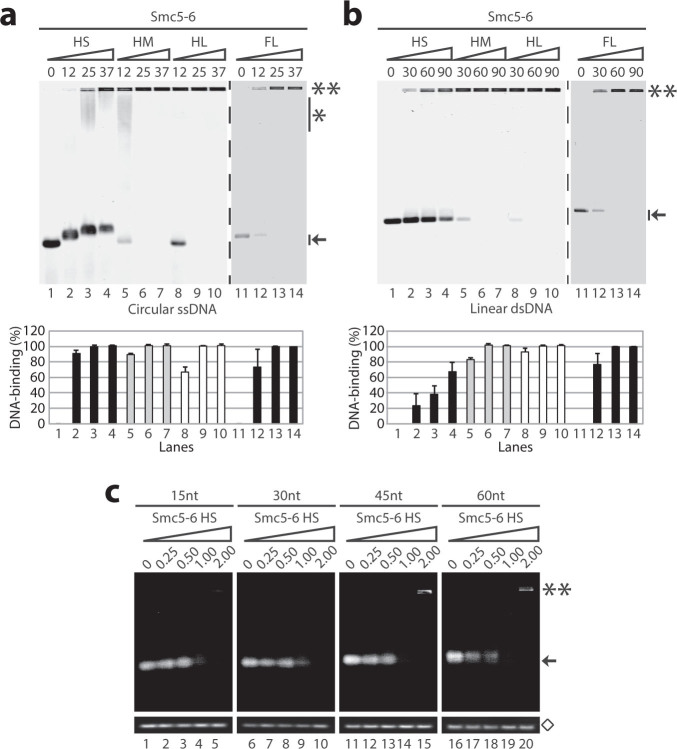
Comparative analysis of the DNA-binding activity of Smc5-6 heterodimer variants. (a) The DNA-binding properties of various Smc5-6 heterodimers were determined by EMSA saturation experiments. The purified proteins were incubated with ssDNA for 30 min at 30 °C and the resulting protein-DNA complexes were resolved by agarose gel electrophoresis, as previously described^22^. The numbers above the gels correspond to the molar ratio of protein over ssDNA in each lane. The double asterisks indicate the position of the protein-DNA complexes; the asterisk with a bar indicates the smear in the gel formed by Smc5-6 heterodimer–DNA complexes, whereas the arrow indicates the position of the free ssDNA. Quantification of DNA-binding is shown in the bar graph below the agarose gel. The unbound ssDNA was quantified in each lane and the data was plotted as a percentage of the protein-DNA complex over free DNA. Each bar is the mean ± standard error of three independent experiments. (b) DNA-binding properties of Smc5-6 hinge fragments and full-length proteins for duplex DNA substrates. The reactions were performed as described above. (c) Length-dependent DNA-binding activity of the Smc5-6 HS heterodimer. The Smc5-6 HS complex was incubated with ss oligonucleotides of different lengths (15, 30, 45 and 60 nt) for 30 min at 30 °C. After incubation, the reactions were loaded on a 2% agarose gel, and the DNA bands were visualized with UV exposure^22^. The numbers above the gel represent the molar excess the molar excess of Smc5-6 HS protein over the ss oligonucleotides. The double asterisks indicate the position of the DNA-bound Smc5-6 HS, whereas the arrow indicates the position of the free DNA in the gel. The diamond indicates the total ss oligonucleotide in each reaction after digestion with a protease and DNA extraction.

Having established a benchmark for the DNA-binding activity of the FL heterodimer, we next conducted additional EMSA experiments to determine whether shorter versions of the Smc5-Smc6 complex can recapitulate the DNA binding activity of the FL heterodimer. We focused our analysis on hinge-containing Smc5-6 fragments since this region is required for dimerization (*i.e*., ATPase head domains are analyzed separately below). As before, a fixed concentration of DNA substrate (ssDNA or dsDNA) was incubated with increasing concentrations of the various Smc5-6 heterodimers. The resulting reactions were loaded on an agarose gel to separate the free DNA from the Smc5-6-bound form. As shown in Fig. 2a, all heterodimers bound to ssDNA with similar affinity, based on the observation that a heterodimer-to-DNA molar ratio of 25 fold is sufficient in all cases to shift the free ssDNA into the gel. However, it is apparent from the different positions of DNA-SMC complexes after electrophoretic separation that the DNA-binding mode is unlikely to be the same for all heterodimers. For instance, the association of the HS construct with DNA generated a novel band migrating above the free DNA but still near the bottom of the gel, irrespective of the protein concentrations used (Fig. 2a; lanes 2–4). This intermediate was not formed in DNA-binding reactions with larger heterodimers. Elongating the length of the coiled-coil region in the hinge fragments progressively slowed the migration of the DNA-bound heterodimers, as evidenced by the formation of a smear near the top of the gel at low heterodimer-to-DNA molar ratios, and the retention of the nucleoprotein complexes at the origin of the gel at high Smc5-6-to-DNA molar ratios (Fig. 2a; lanes 5, 8 and 12). The low mobility of the HM and HL constructs observed in these EMSA experiments is very similar to the behavior of other DNA repair factors acting on ssDNA substrates (*e.g*., Xrs2, RPA, and Rad55-57^33^,^34^,^35^) in comparable gel shift assays. Taken together, these experiments indicate that the Smc5-6 hinge domain contains a *bona fide* ssDNA-binding activity.

We also tested the ability of the heterodimers to bind double-stranded substrates and found that the HS heterodimer bound dsDNA with a much lower affinity than the larger Smc5-6 fragments. Even at 90-fold molar excess relative to DNA, the HS heterodimer did not fully shift the free dsDNA in the gel in EMSA experiments (Fig. 2b; lane 4). In contrast, HM and HL heterodimers exhibited strong dsDNA-binding activity under these conditions (and also at lower protein concentrations; Fig 2b, lanes 6 and 9). Interestingly, HM and HL heterodimers failed to completely bind dsDNA at protein-to-DNA ratios that were sufficient to fully shift ssDNA (compare lanes 6 and 9 in Fig. 2a with lanes 5 and 8 in Fig. 2b). The inability of Smc5-6 HM and HL heterodimers to fully bind dsDNA under conditions that lead to full binding of single-stranded substrates suggests that the low electrophoretic mobility of the heterodimer-ssDNA complexes is not due to unspecific protein aggregation in EMSA experiments. Indeed, from a nucleotide-content standpoint, ss and dsDNA-binding reaction conditions are approximately equivalent (when expressed as molar ratio of DNA substrate relative to Smc5-6 heterodimer; see Methods) and yet only ssDNA associated efficiently with Smc5-6 dimers at protein-to-DNA molar ratios under 30 (compare lanes 3, 6, and 9 in Fig. 2a with lanes 2, 5, and 8 in Fig. 2b). If the binding reactions were driven by non-specific aggregation, one would predict that Smc5-6 should bind equally well to ssDNA and dsDNA under identical chemical (*i.e*., total nucleotide content) conditions. Importantly, the formation of discrete high mobility ssDNA-protein complexes in EMSA experiments involving the HS heterodimer (Fig. 2a; lanes 2–4) demonstrates that this construct contains the minimal domain sufficient for effective ssDNA binding. The presence of additional coiled-coil sequence adjacent to the hinge domain appears necessary to mediate robust binding of Smc5-6 fragments to double-stranded DNA substrates.

### Minimal size of DNA substrates bound by Smc5-6 heterodimers

Since the relative affinity of the HS heterodimer for ssDNA was similar to that of the FL heterodimer, we used the HS heterodimer to determine the minimum ssDNA length required for stable DNA association. To address this question, we used small fluorescent oligonucleotides ranging from 15 to 60 nts in size as substrates in EMSA experiments. Using a similar approach, we previously showed that FL Smc5 and Smc6 monomers can bind stoichiometrically to oligonucleotides of ~45 nts in length^22^,^23^. Consistent with our previous experiments, we observed that substrates of 15 and 30 nts in length interacted weakly with Smc5-6 heterodimers at protein-to-DNA molar ratios ≤ 1 (Fig. 2c; lanes 1–3 and 6–8), and that high concentrations of HS dimer were required to fully shift the DNA away from the unbound position in the gel (*i.e*., arrow in Fig. 2c; lanes 4–5 and 9–10). In contrast, when DNA substrate size was increased to 45 and 60 nts, free DNA was no longer observed at the bottom of the gel at equimolar ratios of substrate and heterodimer (Fig. 2c; lanes 14 and 19). Interestingly, no specific DNA signal was detected in the gel under these conditions, suggesting that Smc5-6 complexes formed multiple nucleoprotein species that are spread out throughout the length of the gel, thereby preventing direct visualization of individual nucleoprotein species (as previously observed^33^,^36^). To confirm this interpretation and to exclude the presence of a contaminating nuclease activity in the HS heterodimer preparation (as a possible explanation for the disappearance of the DNA signal in our EMSA experiments), we treated a fraction of the DNA-binding reaction with a protease and loaded it on a separate gel^33^. After migration, the gel exhibited the same amount of free DNA under all conditions tested (Fig. 2c; diamond band). This observation suggests that the association of the Smc5-6 HS fragment to short ssDNA oligonucleotides creates multiple nucleoprotein species that migrate at different positions after electrophoresis. Consistent with this, further increasing the amount of Smc5-6 heterodimer in the binding reaction saturates the substrates and caused a re-appearance of the 45 and 60 nts ssDNA fragments on top of the gel (Fig. 2c; lanes 15 and 20). Importantly, reversal of the DNA shift upon protease treatment indicates that the low mobility DNA complexes are not due to protein-independent DNA aggregation or covalent linkages created during the binding reaction^37^. Thus, we conclude that the HS heterodimer can bind to DNA substrates as short as 45 nts in length, which fully recapitulates the DNA-binding behavior of the FL Smc5 protein in this regard^22^. Based on its specific affinity and length requirements for ssDNA binding, we consider that the Smc5-6 HS heterodimer contains the minimal region necessary and sufficient to mediate the DNA-binding activity of the native Smc5-6 proteins.

### Smc5-6 heterodimers bind to structured DNA molecules

**Figure 3:**
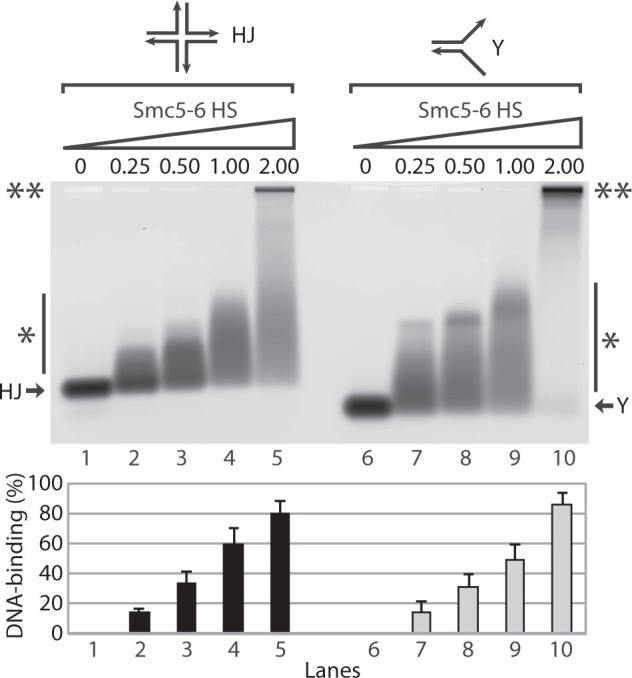
Binding of Smc5-6 heterodimers to structured DNA molecules. The DNA-binding ability of the Smc5-6 HS heterodimer was monitored in EMSA saturation experiments with HJ and splayed Y molecules, as described above. The numbers above the gels correspond to the molar ratio of protein over HJ and splayed Y DNA molecules in each condition. The double asterisks indicate the position of fully-retarded Smc5-6 HS-DNA complexes; the single asterisk next to a vertical bar indicates the positions of partly-retarded nucleoprotein species, and the arrows indicate the positions of free HJ and splayed Y substrates. Quantification of DNA-binding is shown in the bar graph below the gel. The unbound DNA was quantified in each lane and the data was plotted as a percentage of the protein-DNA complex over free DNA. Each bar is the mean ± standard error of three independent experiments.

The involvement of the Smc5-6 complex in DSB repair raises the intriguing possibility that this complex might interact directly with Holliday junctions (HJ) and other structured DNA substrates generated during HR reactions. To address this possibility, we constructed a synthetic HJ and a splayed Y structure (deriving from the HJ) using fluorescently labeled oligonucleotides^38^. Remarkably, incubation of these structured DNA substrates with the Smc5-6 HS heterodimer led to the quantitative formation of retarded nucleoprotein species in EMSA experiments (Fig. 3). The affinity of Smc5-6 for these structured DNA substrates was high, with most DNA structures associating with HS heterodimers at equimolar ratios of protein and DNA substrate. Nucleoprotein complexes formed at low Smc5-6 heterodimer-to-DNA ratios migrated at intermediate positions in the gel (Fig. 3; lanes 2–4 and 7–9), whereas increasing the relative amount of Smc5-6 HS dimer led to the formation of reduced-mobility complexes at or near the origin of the gel (Fig. 3; lanes 5 and 10). Taken together, these observations indicate that Smc5-6 heterodimers efficiently bind to structured DNA molecules typically created during the HR repair process.

### Smc5 monomeric domains: Importance of the coiled-coil and ATPase head regions for DNA-binding activity

**Figure 4:**
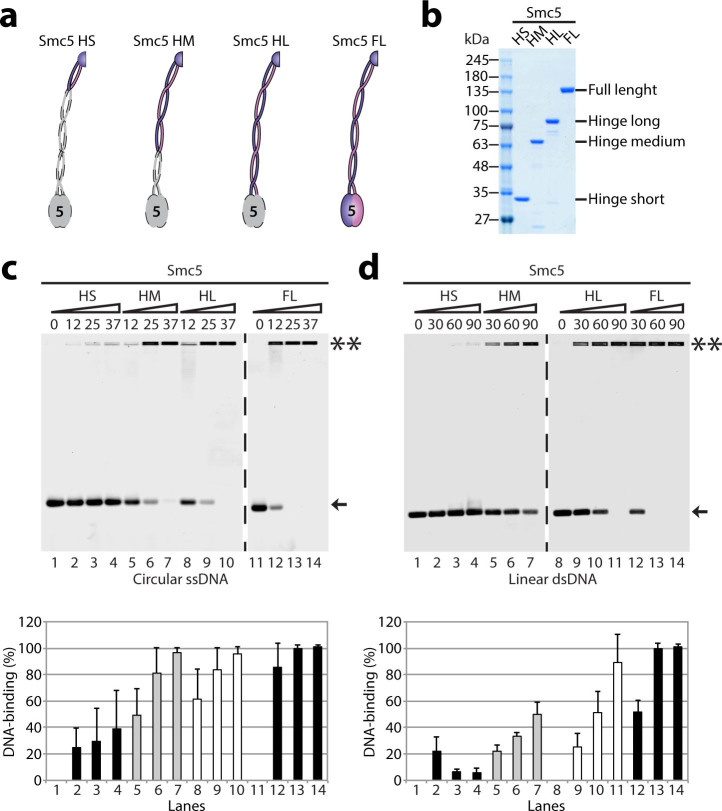
DNA-binding properties of Smc5 monomeric variants. (a) Schematic representation of the Smc5 monomeric variants used in this analysis. The colored regions represent the portion of Smc5 protein included in each construct. (b) Smc5 hinge fragments were purified from bacterial extracts through nickel-NTA affinity chromatography and size exclusion chromatography on Superose 6 or 12 resins. The purity of each construct is shown in the Coomassie-stained gel. (c) The purified Smc5 hinge fragments and full-length proteins were incubated with ssDNA for 30 min at 30 °C as before. The numbers above the gels are the molar ratios of protein over ssDNA. The double asterisks indicate the position of protein-DNA complexes, whereas the arrow indicates the position of free ssDNA. The bar graph below the agarose gel represents the percentage of DNA binding. The unbound ssDNA was quantified as before, and bars in the graph represent the mean ± standard error from three independent experiments. (d) The association of hinge monomer variants and Smc5 full-length protein with dsDNA was tested, as described above.

Having established the DNA-binding properties of Smc5-6 fragments in a heterodimer configuration, we next wanted to know whether the Smc5 monomers would have different DNA-binding characteristics compared to heterodimers. Furthermore, we wanted to study the impact of the ATPase head on the overall DNA-binding activity of the Smc5-6 complex. To achieve this, the functional domains of Smc5 were purified to apparent homogeneity in monomer form (as described above; Fig. 4b), and their DNA-binding properties characterized by EMSA experiments.

All constructs tested bound ssDNA, but to different extents. For instance, the Smc5 HS DNA-binding activity was weak relative to either the Smc5-6 HS heterodimer or the HM and HL monomeric variants of Smc5 (Fig. 4c; lanes 2 to 4). The EMSA experiments also revealed that the DNA binding properties of the HM and HL fragments were comparable, thereby arguing that the minimal region necessary for Smc5 binding to ssDNA as a monomer is located within the HM fragment (Fig. 4c; lanes 5–10). Interestingly, we noticed that we needed ~2-fold more Smc5 HL protein than FL to fully bind the ssDNA (Fig. 4c; lanes 10 and 12), which argues that the HL construct does not contain all the DNA-binding activity of the native protein. Similar results were obtained with dsDNA substrates in EMSA experiments performed with Smc5 monomeric fragments (Fig. 4d). However, we note that even the HM monomer of Smc5 associated somewhat weakly to dsDNA compared to the HL and FL proteins (Fig. 4d; lanes 7 *vs* 11 and 14). Taken together, these results reveal that the major DNA-binding site in the Smc5 monomer is located in the hinge domain and the adjacent coiled-coil region. Increasing the length of the coiled-coil arm beyond that contained within the HM construct did not confer additional ssDNA binding ability, but did have a positive impact on dsDNA association.

**Figure 5:**
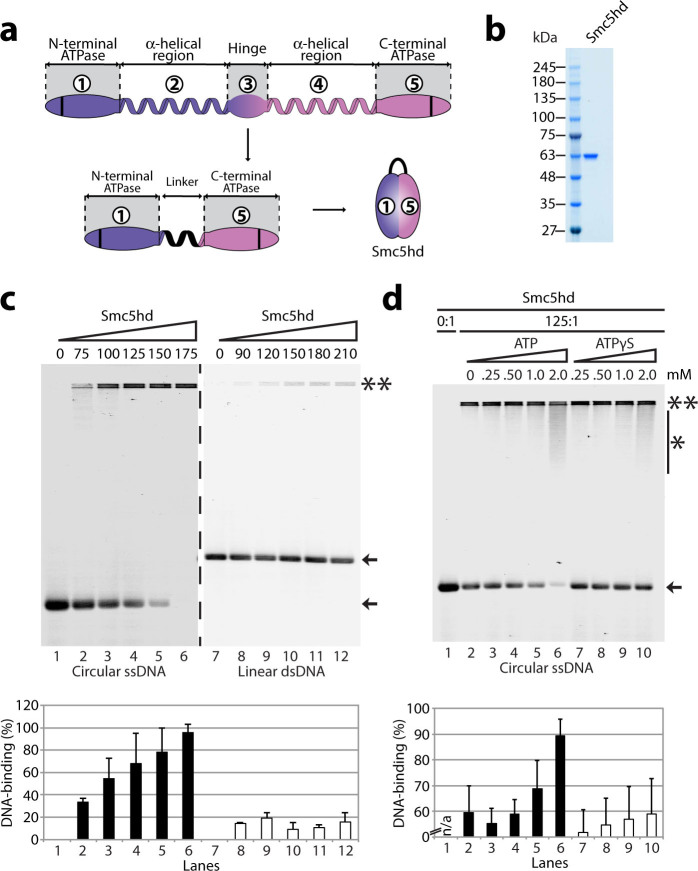
Smc5 ATPase head domain purification and DNA-binding activity. (a) A schematic representation of the domain organization in the unfolded Smc5 protein. To obtain a functional Smc5 ATPase head domain (hd), a small flexible linker of 14 amino acids replaced the central hinge and two α-helical regions. This small linker allowed the two ATPase moieties to fold back on each other and form a complete ATPase head domain^39^. (b) The Smc5hd was purified from yeast extract using nickel-NTA and StrepTrap® affinity chromatography followed by separation via anion exchange on a Q column. The protein purity is shown in the Coomassie-stained gel. (c) The purified Smc5hd was incubated with various types of DNA for 30 min at 30 °C to test its DNA-binding properties, as before. The types of DNA tested were ssDNA (left) and dsDNA (right). The numbers above the gels are the molar ratios of protein over DNA in each lane. The double asterisks indicate the position of the protein-DNA complexes, whereas the arrow indicates the position of free DNA. The bar graph below the agarose gels shows the percentage of DNA binding. The unbound ssDNA was quantified as before, and bars in the graph represent the mean ± standard error from three independent experiments. (d) The effects of ATP or ATPγS on Smc5hd ssDNA binding properties. The purified protein was incubated with ssDNA and various concentrations of ATP or ATPγS for 30 min at 30 °C, and the reactions were processed as previously described. The numbers above the gel indicate the concentration of ATP or ATPγS in each lane. The asterisk with a bar indicates the smear in the gel formed by the Smc5 ATPase head–DNA complexes. The bar graph below the agarose gel represents the percentage of DNA binding. Each bar represents the mean ± standard error from three independent experiments.

The difference in the strength of the ssDNA-binding activity of the FL and HM versions of Smc5 suggests that the native protein might contain more than one independent DNA-binding region. Since the hinge-containing fragments lack the ATPase domain of Smc5, we wondered whether this catalytic domain might also possess DNA-binding activity. To address this possibility, we constructed a monomeric version of Smc5’s bipartite ATPase head domain (Smc5hd) by fusion of the amino- and carboxy-terminal parts of the protein using a short flexible linker, as previously done for the SMC components of the cohesin complex (Fig. 5a)^39^. After overexpression in yeast, the protein was purified using a combination of affinity and ion exchange chromatography steps (Fig. 5b). We then monitored the putative DNA-binding activity of Smc5hd by EMSA experiments. Surprisingly, we observed a specific DNA-binding activity towards ssDNA, but not dsDNA, with purified Smc5hd (Fig. 5c). The protein-to-DNA molar ratio necessary to fully shift the free ssDNA into the gel was 175, which is approximately 7–fold greater than the ratio required when using FL Smc5 protein (Fig. 5c; lane 6). This result prompted us to determine whether ATP might affect Smc5hd DNA-binding activity. To ensure that we can detect both positive or negative effects of ATP on Smc5hd binding to DNA, we used throughout the following series of experiments a Smc5hd:DNA molar ratio that results in partial DNA shifting when ATP is omitted from the EMSA reaction (*i.e*., ~60%, as previously done with the FL protein^22^). Interestingly, supplementing binding reactions with 2 mM ATP increased the DNA-binding activity of Smc5hd by 30% relative to control reactions (Fig. 5d). This stimulatory activity required ATP hydrolysis since no effect was observed when using the non-hydrolyzable ATP analog –ATPγS– in binding reactions (Fig. 5d; lanes 6 *vs* 10). Taken together, these experiments reveal that Smc5 contains at least two independent DNA-binding regions, each located at different ends of the folded SMC rod structure.

### DNA-binding activity of Smc6 monomers

**Figure 6:**
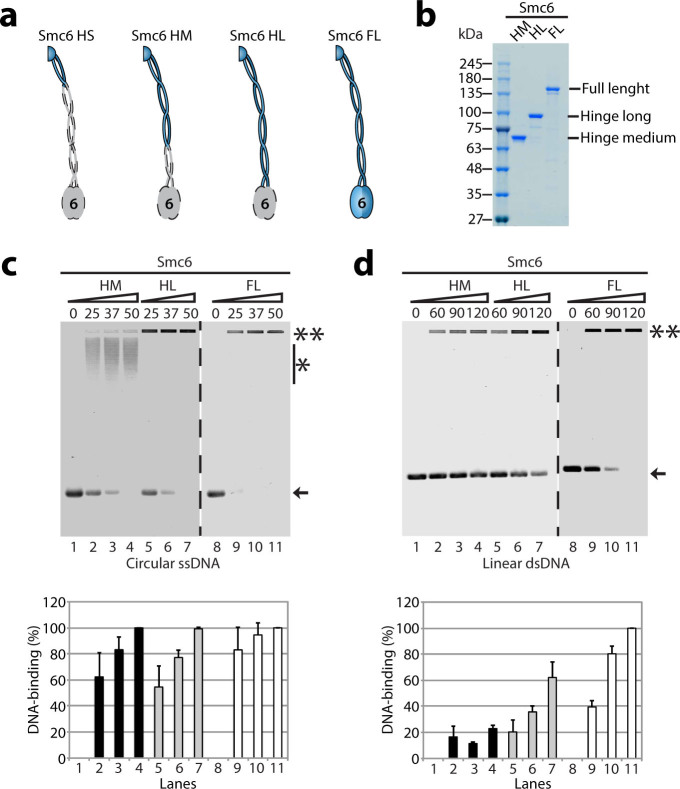
Smc6 fragment purification and DNA-binding activity. (a) Schematic representation of Smc6 fragments and full-length protein used herein. The colored portion shows the parts of Smc6 included in each construct. (b) The Smc6 hinge fragments were purified from bacterial extracts using StrepTrap® affinity chromatography followed by size exclusion chromatography with Superose 6 or 12 resins. The purity of each construct is shown in the Coomassie-stained gel. (c) The purified Smc6 hinge variants and full-length protein were incubated with ssDNA for 30 min at 30 °C to determine their DNA-binding properties, as described above for the Smc5 variants. The bar graph below the agarose gel represents the percentage of DNA binding. Each bar is the mean ± standard error from three independent experiments. (d) The DNA-binding activity of the Smc6 hinge fragments and full-length protein were tested using dsDNA, as before.

We next explored the biochemical properties of Smc6 functional domains in their monomeric forms. Using a similar strategy to that used with Smc5 fragments, several variants of Smc6 were constructed to study the DNA-binding activity of its functional domains (Fig. 6a-b). Except for the Smc6 HS variant, all proteins could be purified to near homogeneity as soluble monomers. Since Smc6 HS was not soluble, we did not include it in our functional analyses.

As before, we used EMSA experiments to determine the DNA-binding activity of the Smc6 variants. Overall, the ssDNA binding affinities were similar when comparing the HM and HL constructs of Smc6 (Fig. 6c; lanes 2–7). However, a clear difference was apparent with regards to the nature of the protein-DNA complex observed in the gel after interaction of HM and HL fragments with ssDNA. Specifically, the HM nucleoprotein complexes migrated as a smear in the EMSA experiment, whereas analogous complexes involving Smc6 HL migrated close to, or at the origin of the gel (Fig. 6c; lanes 3 and 6). This result is similar to that obtained with Smc5 fragments and suggests that extending the length of SMC protein coiled-coil arms causes a more pronounced gel retardation in EMSA experiments. Compared with the FL protein, ~1.5 fold more Smc6 HL protein was needed to fully bind the free ssDNA (Fig. 6c; lanes 6 *vs* 11). This observation lends credence to the notion that Smc6 contains more than one DNA-binding domain. For dsDNA, the binding activity of both HM and HL variants was weak (Fig. 6d; lanes 4 and 7), and significantly more of the HL or HM proteins were required to fully bind dsDNA compared with FL Smc6 (Fig. 6d; lanes 3, 6 *vs* 10).

**Figure 7:**
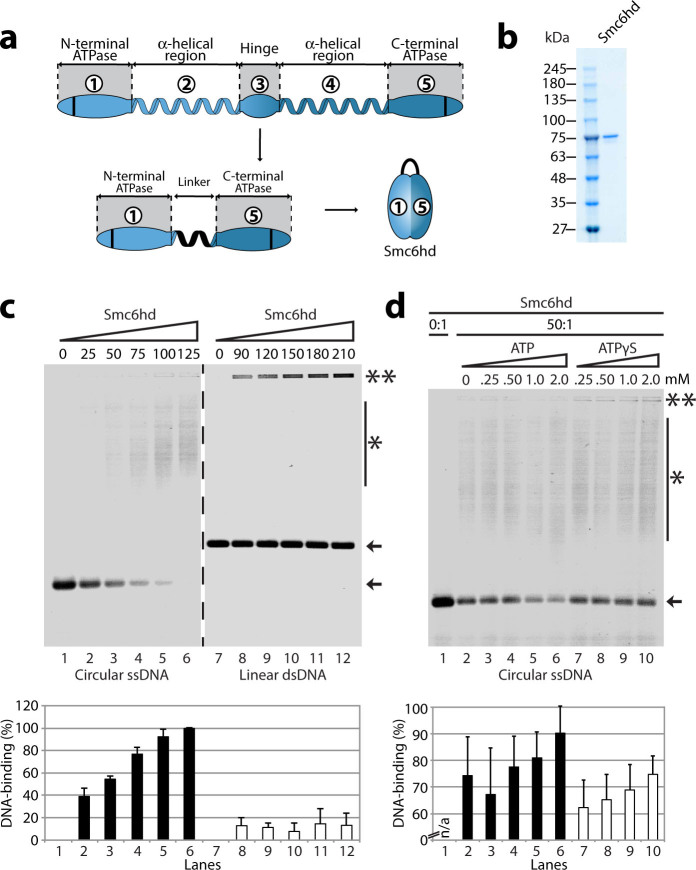
The DNA-binding activity of Smc6 head domain is modulated by ATP hydrolysis. (a) Schematic representation of the unfolded (linear) and folded Smc6 ATPase domain organization. The Smc6hd was obtained using a similar strategy to that showed for Smc5 in Figure 5. (b) The Smc6hd was purified from yeast extract through nickel-NTA, StrepTrap®, and cation exchange chromatography on a SP-sepharose column. The protein purity is indicated in the Coomassie-stained gel. (c) The purified Smc6hd was incubated with various types of DNA for 30 min at 30 °C to test its DNA-binding activity, as described above for Smc5hd. The numbers above the gels are the molar ratio of protein over ssDNA. The double asterisks indicate the position of the most retarded protein-DNA complexes in the gels, the asterisk with a bar indicates the smear in the gel formed by the Smc6hd–DNA complexes, whereas the arrow indicates the position of the free ssDNA. The bar graph below the agarose gel represents the percentage of DNA binding. Each bar is the mean ± standard error from three independent experiments. (d) The effects of ATP or ATPγS on Smc6hd ssDNA binding properties. The purified protein was incubated with ssDNA and various ATP or ATPγS concentrations for 30 min at 30 °C and the reactions were processed as above. To optimize our ability to detect putative effects of ATP in the EMSA assay, we used throughout this series of experiments a Smc6hd:DNA molar ratio that yields a partial DNA shift in absence of ATP (*i.e*., ~60–70% binding under basal conditions). The numbers above the gel indicate the concentration of ATP or ATPγS in each lane. Note that the asterisk with a bar indicates the smear in the gel formed by the Smc6 ATPase–DNA complexes. The bar graph below the agarose gel represents the percentage of DNA binding. Each bar is the mean ± standard error from three independent experiments.

Our previous analysis of FL Smc6 revealed a small, but reproducible, increase in DNA-binding activity when the protein was incubated with ATP^23^, thereby suggesting a potential role for the ATPase domain of the protein in DNA-binding activity. To test this hypothesis, we overexpressed the Smc6 ATPase head domain in yeast (Smc6hd, Fig. 7a), and we purified the protein to greater than 95% homogeneity (Fig. 7b). As predicted, Smc6hd bound ssDNA extensively in EMSA experiments, but interacted weakly with double-stranded substrates under similar binding conditions (Fig. 7c). Approximately 3,3 fold more Smc6hd was necessary to fully bind free DNA when compared to the FL protein. Supplementing 2 mM ATP to the DNA-binding reaction stimulated ssDNA binding by 20% (Fig. 7d; lanes 2 and 6), and substitution of ATP with ATPγS abolished the stimulation (Fig. 7d; lanes 7–10). Taken together, these results indicate that Smc6 is able to associate with DNA substrates via both its ATPase and hinge domains, and that this is a conserved property of the SMC components of the Smc5-6 complex.

## Discussion

This study provides critical insights into the mode of action and structural requirements for effective DNA binding by the Smc5-6 complex. Using a series of functional domain fragments, we reveal how the Smc5 and Smc6 proteins can interact with their DNA substrates via two distinct DNA-binding domains on each SMC molecule. The combinatorial use of these individual domains in Smc5-6 complexes is likely to confer multivalent DNA binding properties and highly resistant DNA association *in vivo*. Consistent with this view, we show that formation of the Smc5-Smc6 heterodimer increases the affinity of the resulting complex specifically for dsDNA substrates. Finally, we present evidence that suggests a putative role for ATP hydrolysis in the regulation of SMC protein association with DNA. Collectively, these discoveries have major implications on our understanding of how the Smc5-6 complex interacts with chromosomal DNA substrates in living cells, and how these interactions might contribute to effective DNA repair and chromosome segregation *in vivo*.

From a functional standpoint, the domain-specific analysis of Smc5-6 presented herein has revealed the existence of at least two DNA-binding regions in each of these proteins. The first region is located in the hinge domain and adjacent coiled-coil arm sequences, whereas the second DNA-binding region is located in the ATPase head domain of the protein. This bivalent DNA-binding activity is not typical of all SMC proteins. Indeed, with the notable exception of *T. maritima* SMC^40^, all bacterial SMC members bind DNA substrates via either their ATPase head or hinge domains, but not both. For instance, the two most studied prokaryotic SMC proteins –the MukB protein from *E. coli* and *Bacillus subtilis* SMC (BsSMC)– have been shown to contain a single DNA-binding domain located at the junction between the ATPase and coiled-coil regions^41^ and at the hinge domain, respectively^42^. The situation is more complex in the case of eukaryotic SMC proteins. Smc1 and Smc3, the SMC components of the cohesin complex, can bind to DNA via the extreme C-terminal portion of their ATPase domains^43^,^44^ as well as through their hinge domains^45^,^46^,^47^, whereas the DNA-binding activity of the SMC subunits of the condensin complex appears to reside primarily in their hinge domains^27^,^46^. Interestingly, both the hinge- and ATPase head-mediated DNA interactions of cohesin’s SMC components show much higher affinity for dsDNA than for ssDNA^43^,^44^,^45^, a preference that is opposite to that of the homologous domains in Smc5 and Smc6. This may reflect the primary function of cohesin in mediating sister chromatid cohesion, which does not involve ssDNA intermediates associating typically with DNA repair reactions. Thus, with regards to its high affinity for ssDNA substrates, the Smc5-6 complex resembles more the Smc2 and Smc4 components of condensin and of bacterial SMC complexes^46^,^48^,^49^. Another key difference between the cohesin and Smc5-6 complexes is the fact that Smc1 and Smc3 hinge regions require dimerization to mediate DNA binding^45^, while Smc5 and Smc6 hinges bind DNA substrates with high affinity both as monomers and as a heterodimer. The ability of Smc5-6 to bind DNA segments as a monomer is consistent with the fact that Smc5 exerts some of its functions in the absence of Smc6 and other components of the complex during mitosis^28^.

It is worth mentioning that ATP seems to have distinct effects on Smc5 DNA-binding activity depending on whether the whole protein or only the ATPase head domain are analyzed. The presence of dual DBDs in native Smc5 may explain this difference. Indeed, it has been previously noted that binding of DNA substrates near the ATPase head domain of Rad50 –a SMC-like repair factor– can induce mesoscale conformational changes at the other end of the molecule (i.e., in a region corresponding to the hinge domain in Smc5-6)^50^. In light of this, it is possible that the ATPase head domain of Smc5 might allosterically regulate the DNA-binding activity of its hinge domain. In this context, one would expect that the DNA-binding behavior of the Smc5hd fragment would show only part of the full DNA-binding response of the native molecule in the presence of ATP. Separate from this, it is also possible that the different types of DNA-binding assays used to monitor the effect of ATP on Smc5hd and native Smc5^22^ could explain part of the distinct responses of these proteins to the presence of nucleotides. Additional studies will be required to determine whether the ATPase head domain of Smc5 can effectively regulate at distance the DNA-binding activity of its hinge domain, and what would be the potential impact of this mode of regulation on Smc5-6 complex function.

Our biochemical characterization of Smc5-Smc6 heterodimers has several important implications for DSB repair reactions. During DSB repair by homologous recombination, one key step is the search for homologous DNA sequences in the genome using Rad51-ssDNA filaments^51^. However, the RPA complex must bind ssDNA before this can take place. The affinity of RPA for ssDNA is between 10^−9^ to 10^−11^ M, whereas that of Rad51 for the same substrate is 3×10^−7^ M^52^,^53^. Thus, based solely on substrate affinity, the Rad51 protein might not be able to displace RPA from ssDNA, which is why Rad51 requires mediators to assemble into a filament in the presence of RPA-bound ssDNA^54^. In comparison, the Smc5-6 heterodimer affinity for ssDNA is approximately 4x10^−8^ M, which is lower than that of RPA for the same substrate, but is also significantly higher than that of Rad51. Thus, the role played by the Smc5-6 complex in antagonizing homologous recombination at the rDNA/nucleolus^14^ may be explained by a competitive advantage over Rad51 for its association with ssDNA substrates at this locus. Moreover, the nucleolar exclusion of a known mediator of Rad51 activity, the Rad52 protein^14^, suggests that binding and filament formation on single-stranded rDNA substrates might rely more on Rad51 intrinsic affinity for this substrate, a scenario that would favour Smc5-6 complex association to ssDNA *in vivo*^55^. It is still unclear whether there is also a competitive relationship for ssDNA binding between RPA and the Smc5-6 complex during DSB repair reactions. It is conceivable that the Smc5-6 complex might require mediators, as in the case of Rad51, to displace RPA and bind to ssDNA substrates in the genome. A possible candidate for this function is the Nse5-6 complex, which interacts with the hinge domains of Smc5 and Smc6^56^, and might facilitate entry of the DNA substrate between the arms of Smc5 and Smc6 in a manner analogous to DNA entry into the cohesin ring complex^47^. At this point, we favor a non-competitive relationship between RPA and Smc5-6 complex binding to DNA based on the fact that, at saturation, RPA binds ssDNA every 90 to 100 nts^57^. Since RPA covers only 30 nts of ssDNA upon binding^52^, this leaves approximately 60 to 70 nts of the substrate exposed between two RPA complexes. Our experiments revealed that tracks of 45–60 nts ssDNA are sufficient for stable binding of the Smc5-6 complex. Thus, it is conceivable, if not likely, that both RPA and Smc5-6 complexes would bind simultaneously to the same ssDNA fragments during DNA repair reactions. In this regard, it would be interesting to conduct *in vivo* experiments using the *rfa1-t11* mutant^58^–an allele of RPA with altered ssDNA-binding properties– to determine if this condition alters Smc5-6 complex localization to ssDNA lesions.

Loss of Smc5-6 complex activity leads to the formation of unresolved links between homologous chromosomes, incomplete chromosome replication, and aberrant mitotic chromosome formation^15^,^29^,^59^. If these abnormal chromosome structures persist until anaphase, they are likely to generate gross chromosome instability, a hallmark of cancer^60^. In this regard, it is interesting to note that the Smc5-6 complex is unloaded from chromosomes in late mitosis^29^,^30^,^31^,^32^ and that this process is apparently associated with the dissociation of the Smc5 subunit from the Smc5-6 complex^28^. Our results indicate that dissociation of Smc5 from the other components of the complex will likely affect their affinity for dsDNA substrates. Indeed, we have shown that Smc5-6 heterodimers bind more strongly to dsDNA than either SMC components in their monomeric form. The unloading of the Smc5-6 complex from mitotic chromosomes may be driven by a loss of intrinsic affinity for duplex DNA in the mitotic form of the Smc5-6 complex. One reason why this dissociation process would be important for cells is because the multiple DNA-binding domains of Smc5-6 components might create intermolecular/non-sister chromatid linkages^21^ that could impede chromosome segregation in anaphase. Dissociation of Smc5 from Smc6, and the accompanying reduction in the intrinsic affinity of the resulting complexes for chromosomal dsDNA, would thus provide an elegant mechanism to ensure effective chromosome partition during mitosis. This hypothesis is consistent with the formation of persistent DNA bridges in anaphase cells with misregulated Smc5-6 complex components^29^,^61^. Testing this idea will require the identification of the trigger that is responsible for the mitosis-specific dissociation of Smc5 from the rest of the complex, an objective that will be the focus of future work.

## Methods

### Plasmids for Smc5-6 overexpression

The boundaries of Smc5 and Smc6 head domains, coiled-coil and hinge regions were determined using PSIPRED^62^. DNA fragments encoding the functional regions of Smc5 or Smc6 were amplified by PCR and cloned into pETDuet-1, pET28a, pET41a or pET30a vectors (Novagen) for expression in *Escherichia coli*. The Smc5 fragments were expressed as fusion proteins with a carboxy-terminal nona-histidine tag, whereas Smc6 fragments were fused at their amino-terminus with the Strep-TagII sequence. The bipartite ATPase head domains of Smc5 and Smc6 were amplified by PCR, and both parts were connected during the amplification procedure with a primer encoding a 14-residue flexible linker, as done previously for Smc1 and Smc3^39^. These constructs were then fused to a tandem affinity purification tag (3xStrep-TagII and 9xHIS; STH) at their carboxy-termini and subcloned downstream of the *GAL1* promoter in a 2µ *URA3* *leu2-d* containing plasmid for expression in *S. cerevisiae*. The detailed amino acid positions included in each protein fragment used in this study are listed in Table 1.

### Protein expression and purification in *E. coli*

All hinge domain-containing proteins were expressed in Rosetta 2 DE3 pLys cells (Novagen)^63^. Bacterial cultures were grown at 37 °C to an A_600_ of 0.6 and induced to express recombinant proteins by addition of 1 mM isopropyl β-thiogalactopyranoside for 6 h at 20 °C. Cells were harvested and lysed in buffer A (50 mM K_2_HPO_4_/KH_2_PO_4 _pH 8.0, 50 mM Tris-HCl, pH 8.0, 500 mM NaCl, 10% glycerol, 2 mM 2-mercapthoethanol (2-ME), 0.2% Triton X-100 supplemented with phosphatase and protease inhibitor cocktail set IV (EMD) and 100 μg/mL lysozyme). After 30 min of incubation on ice, cells were sonicated (3 pulses of 10 sec at output level 4 using a Misonix Sonicator 3000). The crude lysates were centrifuged at 50,000 g for 30 min at 4 °C. Soluble proteins were collected and incubated with Ni-NTA agarose resin (Qiagen) for 1 h. The resin was washed with 10 column volumes (CVs) of buffer B (25 mM K_2_HPO_4_/KH_2_PO_4_, pH 8.0, 500 mM NaCl, 10% glycerol, 0.2% Tween 20, 2 mM 2-ME) supplemented with 20 mM imidazole and eluted with 3 CVs of NS buffer supplemented with 500 mM imidazole. The eluates were then diluted 3-fold with buffer NS (25 mM K_2_HPO_4_/KH_2_PO_4_, pH 8.0, 750 mM NaCl, 5% glycerol, 0.7% Tween 20) before loading on a *Strep*-Tactin® column (GE Healthcare). The column was washed with 10 CVs of NS buffer and eluted with 5 CVs of NS buffer supplemented with 2 mM desthiobiotin. The eluates were concentrated by ultrafiltration using Amicon Ultra filtration units (10K NMWL; Millipore) and further purified by size exclusion chromatography on a Superose 6 10/300 column (GE Healthcare) in buffer C (25 mM K_2_HPO_4_/KH_2_PO_4_, pH 8.0, 500 mM NaCl, 10% glycerol, 0.2% Tween 20, 2 mM 2-ME, 1 mM EDTA). The final fractions that contained purified proteins were concentrated to ~2 mg/mL using Amicon Ultra filtration units (10K NMWL; Millipore), frozen on dry ice and stored at −80 °C. To purify Smc5 monomeric fragments, we used the same procedure with the following modifications. Proteins were purified by single-step Ni-NTA chromatography, buffer A contained 150 mM NaCl, and buffer C contained 750 mM NaCl. To purify Smc6 monomeric fragments, we used the same procedure as described above with the following modifications. Proteins were purified using single-step *Strep*-Tactin® chromatography, buffer A contained 150 mM NaCl, and buffers B and C contained 750 mM NaCl.

### Protein expression and purification in yeast

The Smc5 head domain was expressed in yeast strain D3596 using standard procedures^64^. Protein expression was induced for 4 h with galactose (2% final) in a 1 L yeast culture. The Smc5 head domain-overexpressing cells were resuspended in 4 mL of lysis buffer (100 mM K_2_HPO_4_/KH_2_PO_4_, pH 8.0, 50 mM Tris-HCl pH 8.0, 50 mM NaCl, 5% glycerol supplemented with phosphatase and protease inhibitor cocktail set IV (EMD)) and lysed as previously described^64^. After lysis, salt was adjusted to 1 M NaCl, glycerol was adjusted to 5%, and the lysate was centrifuged at 16,500 g at 4 °C for 15 min. The soluble proteins were collected, and the lysate pH was adjusted to 8.0. The lysate was then diluted 2-fold with buffer D (25 mM K_2_HPO_4_/KH_2_PO_4_, pH 8.0, 500 mM NaCl, 5% glycerol and 0.7% Tween 20) and incubated with Ni-NTA agarose resin (Qiagen) for 1 h. The resin was washed with 10 CVs of buffer D supplemented with 20 mM imidazole and eluted with 3 CVs of buffer D supplemented with 250 mM imidazole. The eluate was then diluted 10-fold with buffer Q1 (Tris-HCl at pH 8.0, 10% glycerol, 0.7% Tween-20, 2 mM 2-ME, 1 mM EDTA) and passed through a Q-Sepharose FF column® (GE Healthcare). The flow-through was then loaded on a *Strep*-Tactin® column (GE Healthcare), washed with 10 CVs of buffer E (25 mM K_2_HPO_4_/KH_2_PO_4_, pH 8.0, 750 mM NaCl, 15% glycerol, 0.2% Tween 20, 2 mM 2-ME, 1 mM EDTA) and eluted with 5 CVs of buffer E supplemented with 2 mM desthiobiotin. The final fractions containing purified proteins were concentrated to ~1 mg/mL with Amicon Ultra filtration units (10K NMWL; Millipore), frozen on dry ice and stored at −80 °C.

For the Smc6 ATPase head domain, overexpression and cell lysis were performed exactly as described above using yeast strain D3862. After centrifugation, the soluble proteins were collected, and the lysate pH was adjusted to 8.0. The lysate was then diluted 2 fold with buffer F (25 mM K_2_HPO_4_/KH_2_PO_4_, pH 8.0, 10 mM Tris-HCl at pH 8.0, 500 mM NaCl, 10% glycerol, 0.2% Tween 20, 2 mM 2-ME) and incubated with Ni-NTA agarose resin (Qiagen) for 1 h. The resin was washed with 10 CVs of buffer F supplemented with 20 mM imidazole and eluted with 3 CVs of buffer F supplemented with 500 mM imidazole. The eluate was then diluted 3-fold with buffer F and loaded on a *Strep*-Tactin® column (GE Healthcare), washed with 10 CVs of buffer F and eluted with 5 CVs of buffer F supplemented with 2 mM desthiobiotin. Next, the eluate was diluted 10-fold with buffer SP- (50 mM K_2_HPO_4_/KH_2_PO_4_, pH 8.0, 10% glycerol, 0.2% Tween 20, 2 mM 2-ME) and loaded on an SP-Sepharose FF column® (GE Healthcare), washed with 10 CVs of buffer SP1 (buffer SP- containing 50 mM NaCl) and eluted with a linear gradient of 10 CVs of buffer SP1 and buffer SP2 (buffer SP- containing 1 M NaCl). The protein was eluted in a fraction containing approximately 130 mM NaCl. The final fractions containing purified proteins were concentrated to ~0.5 mg/mL with Amicon Ultra filtration units (10K NMWL; Millipore), frozen on dry ice and stored at −80 °C.

### Reconstitution of full-length Smc5-6 heterodimers

To reconstitute the FL Smc5-6 heterodimer, individual subunits were expressed and purified as previously described^22^,^23^, with the exception that we used a StrepTrap® column (GE Healthcare) instead of StrepTactin®. After the StrepTrap® chromatograpy, we mixed 5 mL of the Smc6 eluate with 150 µL of the Smc5 eluate, and supplemented this mixture with phosphatase and protease inhibitor cocktail set IV (EMD). After overnight incubation at 4 °C, the proteins were concentrated via ultrafiltration using Amicon Ultra filtration units (10K NMWL; Millipore) and complexes were separated from monomeric subunits by size exclusion chromatography on a Superose 6 10/300 column (GE Healthcare) in buffer G (25 mM K_2_HPO_4_/KH_2_PO_4_, pH 8.0, 750 mM NaCl, 15% glycerol, 0.2% Tween 20, 2 mM 2-ME, 1 mM EDTA). The final fractions containing purified proteins were concentrated to ~150 ng/mL with Amicon Ultra filtration units (10K NMWL; Millipore), frozen on dry ice and stored at −80 °C.

### DNA binding experiments

Plasmid substrates used in DNA-binding experiments were phiX174 (ssDNA substrate; 5386 bp) and EcoRI-digested pBluescript II KS+ (dsDNA substrate; 2961 bp). At a given molar fold-excess of Smc5 heterodimer-to-DNA, one may consider the nucleotide content of ss and dsDNA-binding reactions to be similar since the ssDNA substrate is approximately twice the size of the dsDNA substrate, but the later contains twice the nucleotide content per unit of length because of its double-stranded nature. The DNA binding properties of Smc5-6 proteins were determined by electrophoretic mobility shift assay, essentially as described previously^22^,^23^. HJ and splayed Y substrates were assembled as described previously, with minor modifications^38^. Four complimentary oligonucleotides (HR1, HR2, HR3 and HR4; for HJ) and 2 partially complimentary oligonucleotides (HR1 and HR2; for splayed Y) were annealed in 10 mM Tris-HCl (pH 8.5) at a concentration of 5 µM. The mixtures were incubated for 2 min at 95 °C, followed by 10 min at 65 °C, 10 min at 37 °C, and 10 min at room temperature (in total volume of 100 µl). The entire mixtures were separated on a 2% TAE agarose gel, and the corresponding bands (HJ and Y) were excised from the gel. DNA was recovered using a strandard gel extraction procedure (QIAquick® gel extraction; Qiagen). An aliquot from this final sample was run on 10% native acrylamide gel to confirm the purity and integrity of the HJ and Y DNA structures. Binding of Smc5-6 HS to structured DNA molecules was determined by EMSA saturation experiments in Holliday junction buffer (10 mM Hepes pH 7.5, 50 mM NaCl, 7 mM MgCl_2_, 20% glycerol, and 2 mM 2-ME). Protein-DNA complexes were visualized after electrophoresis using the 6-FAM fluorophore conjugated to the 5’ end of the HR1 and HR2 oligonucleotides. ssDNA oligonucleotides used in EMSA experiments were as described previously^22^. Treatment of nucleoprotein complexes with proteinase K was performed as previously described with minor modifications^33^.

## Author Contributions

M.-A.R. designed, performed, and analyzed experiments presented Figures 1, 2, 4–7; T.D. designed, performed, and analyzed the experiments presented in Figure 3; M.-A.R., T.D., and D.D. wrote the manuscript.

## Additional Information

**How to cite this article**: Roy, M.-A., Dhanaraman, T. & D’Amours, D. The Smc5-Smc6 heterodimer associates with DNA through several independent binding domains. *Sci. Rep.* **5**, 9797; doi: 10.1038/srep09797 (2015).

## Figures and Tables

**Table 1:** Plasmids used in this study

Protein	Amino acid position	Vector backbone
**Smc5 HS**	428–675	pETDuet-1/pET-28a
**Smc5 HM**	300–803	pETDuet-1/pET-28a
**Smc5 HL**	215–885	pET-28a
**Smc5 hd**	1–230 and 910–1093	YEpFAT4
**Smc5 FL**	1–1093	YEpFAT4
**Smc6 HS**	486–732	pETDuet-1/pET-41a
**Smc6 HM**	350–868	pETDuet-1/pET-41a
**Smc6 HL**	260–970	pET-30a
**Smc6 hd**	1–280 and 820–1114	YEpFAT4
**Smc6 FL**	1–1114	YEpFAT4
